# Artificial Lipid Droplets: Novel Effective Biomaterials to Protect Cells against Oxidative Stress and Lipotoxicity

**DOI:** 10.3390/nano12040672

**Published:** 2022-02-17

**Authors:** Pengxiang Zhao, Yi Jin, Xiang Wu, Jin Huang, Lupeng Chen, Yanjie Tan, Hong Yuan, Jian Wu, Zhuqing Ren

**Affiliations:** 1 Key Laboratory of Agriculture Animal Genetics, Breeding and Reproduction of the Ministry of Education, College of Animal Science, Huazhong Agricultural University, Wuhan 430070, China; pengxiang@webmail.hzau.edu.cn (P.Z.); Jinhongyi@mail.hzau.edu.cn (Y.J.); Wx1078724218@163.com (X.W.); huangjin@webmail.hzau.edu.cn (J.H.); chenlupeng@webmail.hzau.edu.cn (L.C.); 620036@sdnu.edu.cn (Y.T.); wujian@mail.hzau.edu.cn (J.W.); 2 Key Laboratory of Pesticide and Chemical Biology of Ministry of Education, College of Chemistry, Central China Normal University, Wuhan 430079, China; yuanhong@mail.ccnu.edu.cn; 3 Hubei Hongshan Laboratory, Wuhan 430072, China

**Keywords:** artificial lipid droplets, biomaterials, lipotoxicity, ROS, Plin5, cell stress

## Abstract

Lipid droplets (LDs) play an important role in the regulation of cellular stress. This suggests LDs can be applied as safe and effective biomaterials to alleviate cellular stress and lipotoxicity. Here, we constructed a convenient method to generate stable and pure artificial lipid droplets (aLDs). aLDs can maintain their biological function by incubating LD-associated proteins or organelles in vitro. It was validated that perilipin-coated aLDs could be uptaken by cells, significantly reducing hydrogen peroxide-induced reactive oxidative species (ROS) and alleviating cellular lipotoxicity caused by excess fatty acid. Our work demonstrated a direct role of LDs in regulating cellular stress levels, providing methods and potential value for future research and medical applications of LDs.

## 1. Introduction

Lipid droplets (LDs) have a unique structure, consisting of a neutral lipid core and a monolayer of phospholipid membranes encapsulated by various proteins [1]. LDs have been recognized as organelles with important functions in cellular metabolism [2]. In addition to lipid storage, LDs play a critical role in resisting stress and maintaining intracellular homeostasis under stressful conditions, such as nutrient deficiency or reactive oxidative species (ROS) [3,4]. It has been shown that LDs can protect cells from cellular damage caused by ROS [5,6], such as transferring oxidized lipids from cell membranes to themselves [7,8]. Excessive lipid uptake can also trigger cellular lipotoxicity [9]. Cells mitigate lipotoxicity by activating lipid synthesis pathways to convert fatty acids into neutral lipids such as triglycerides and sterols, and then store them in LDs [10]. The key protein which regulates oxidative stress is named Plin5. Under basal conditions, Plin5 can directly interact with adipose triglyceride lipase (ATGL) to inhibit the conversion of palmitic acid to triacylglycerol (TAG) and reduce the utilization of palmitate by mitochondria. However, the effects are contrary under lipid stimulation [11,12,13]. Meanwhile, Plin5 can mediate monounsaturated fatty acids (MUFA) signaling to control sirtuin 1(SIRT1)/PPARG coactivator 1 alpha (PGC-1α) and promote the transport of unsaturated fatty acids [14]. All these results suggest that LDs play an important role in regulating cellular stress and maintaining cellular homeostasis. LDs are the accumulation or action site for some drug molecules. The lipid-soluble antibiotic (Bedaquiline) accumulates in LDs of patient macrophages and enhances its antibacterial effect against mycobacterium tuberculosis [15]. This suggests that drugs can accumulate in LD and be transferred to pathogens when they are consumed. The direct evidence is lasonolideA (LasA), a macrocyclic endolipid antibiotic which accumulates in LDs. LasA acts through its hydrolysis product LasF which is hydrolyzed by the LDs surface protein lipid droplet associated hydrolase (LDAH). Inhibition of lipid production diminishes LasA efficacy by 2-3-fold [16]. Recent studies have shown that cell-derived LDs can act as controllable and biocompatible carriers to deliver anticancer drugs and significantly inhibit tumor growth [17]. These studies revealed the role of LDs in intracellular drug metabolism and built a LDs-based drug delivery system.

The highly dynamic feature (differences in proteins and lipid components) of LDs still mean we lack a comprehensive understanding of LDs. The LDs size, location, lipid and protein composition rapidly altering with environmental stimuli and cellular state. The size ranges from 40 nm~1 μm, and the size in adipocytes can even reach 100 μm [18]. Some researchers have tried to isolate LDs from cells, but LDs proteomic data always contain other impurities, such as endoplasmic reticulum (ER) and mitochondria, which limits researchers’ ability to accurately analyze LD function [19,20]. In recent years, synthetic biology has developed rapidly and has been able to synthesize cell-like structures with ATPase activity [21]. An in vitro experimental system for LDs has been constructed and has identified the function of proteins such as microorganism lipid droplet small protein (MLDS) and ATGL [22,23,24]. A LDs-like structure is obtained by injecting triglycerides into a giant unilamellar vesicle (GUV), which confirms that surface tension affects the directionality of lipid droplet growth [25]. Microfluidics has also been applied to LDs in studies to identify the role of coat complex subunit beta 1(COPI) in LDs protein transport [26]. However, those methods have not been widely applied in LDs research because of complex processes, difficult techniques and too many impurities.

Inspired by these research projects, we suppose that LDs can be applied as safe and effective biomaterials to alleviate cellular stress and lipotoxicity. Here, we constructed a method to generate stable and pure artificial lipid droplets (aLDs). Perilipin-coated artificial LDs can be taken up by cells and significantly reduce cellular ROS levels and mitigate cellular lipotoxicity. This artificial biomaterial contains good stability in the cellular environment, providing potential value for LDs research and medical applications.

## 2. Material and Methods

### 2.1. Materials

1,2-Di(9z-octadecenoyl)-sn-glycero-3-phosphocholine (DOPC), 1,2-di(9z-octadecenoyl)-sn-glycero-3-phosphoethanolamine (DOPE), 1,2-dioctadecanoyl-sn-glycero-3-phosphocholine (DSPC), 1,2-dipalmitoyl-sn-glycero-3-phosphocholine (DPPC), triacylglycerol (TAG) and cholesteryl oleate (CO) were purchased from TCI (Tokyo, Japan). Phosphatidylinositol (PI) and phosphatidic acid (PA) were purchased from Aladdin (Shanghai, China). Lipid Tox red (H34477), BODIPY493/503 (493/503) and colloidal blue staining kits were obtained from Invitrogen. Flp was obtained from Liu’s groups. The acquired microfilaments were from cytoskeleton. DGAT1 inhibitor (A922500) and DGAT2 inhibitor (PF-06424439) were purchased from MedChemExpress. The Recombinant Apolipoprotein B (APOB) protein was purchased from Cloud-Clone (#RPC003Hu01, CLOUD-CLONE, China).

### 2.2. Construction of Nanolipid Particles

Triglycerides (300 mg) and DOPC (30 mg) were added to 10 mL ethanol solution and heated in a water bath until completely dissolved. Then the solution was completely mixed by vortexing for 30 s to obtain the organic phase. We took 30 mL of the solution containing 0.2% Tween 80 as the aqueous phase. The organic phase was slowly added to the aqueous phase and stirred on a magnetic stirrer to complete the volatilization of ethanol (400 rpm, 4 h). Then, the solution was quickly cooled in an ice water bath, and NLPs were obtained. The NLPs were collected for further morphological, biochemical, and functional analyses. The NLP sizes were determined by dynamic light scattering (DLS, Delsa Nano C-Particle Analyzer, Beckman), and the NLP concentration was measured by optical density at 600 nm (OD 600) using a Perkinelmer Enspire [22]. In this study, NLPs were obtained according to this method without special modifications.

### 2.3. Cell Culture

The HepG2 and Hela cell line was purchased from the Type Culture Collection of the Chinese Academy of Sciences (Wuhan, China). HepG2 and Hela cells were cultured in Dulbecco’s modified Eagle’s medium (DMEM; HyClone, Logan, UT, USA) with 10% fetal bovine serum (FBS; #SH30396.03, HyClone, Ottawa, Canada), 100 unit/mL penicillin, and 100 μg/mL streptomycin in dishes at 37 °C in a humidified atmosphere with 5% CO_2_. For palmitic acid (PA) treatment, 40 mM PA-NaOH and 40% FA-free bovine serum albumin (BSA) medium were prepared, and both solutions were heated in a 75 °C water bath for 30 min. Finally, the solution was mixed. We obtained 200 μM PA medium from adding PA-BSA mixture to the cell medium at 1:99 (*v*:*v*). the cells washed three times by PBS and 1 mL PA medium was added to the well and cultured for 12 h. For oleic acid (OA) treatment, 20 mM OA-phosphate buffer saline (PBS) mixture and 20% FA-free bovine serum albumin (BSA) medium were prepared. The medium was mixed after both media were heated in a 70 °C water bath for 30 min. We obtained 200 μM OA medium by adding OA-BSA mixture to the cell medium at 1:49 (*v*:*v*). We washed the cells three times with PBS and 1 mL OA medium was added to the well and cultured for 12 h. The cells were then either seeded on slides, or on plates that had been washed three times using PBS. Then, 1 mL OA medium was added to the well, and the cells were cultured for 12 h. For H_2_O_2_ treatment, we incubated cells with 200 µM H_2_O_2_ 6 h.

### 2.4. Cellular Uptake

To obtain fluorescently labeled aLDs (Rhod-PE aLDs), 2% rhodamine-labeled phosphatidylethanolamines (Rhod-PE) was mixed in DOPC to generate NLPs as described above. We incubated 10 μg Plin5 protein with NLPs at 37 °C for 1 h, then the mixture was centrifuged in 12,000× *g* (RT) for 3 min, and the Plin5-coated aLDs were collected from the upper band. For the cellular uptake experiment, Hela cells were incubated with aLDs for 4 h, followed by organelles staining and confocal microscopy imaging.

### 2.5. Reactive Oxygen Species (ROS) Measure

An ROS assay kit (#S0033S, Beyotime Biotechnology, Nanjing, China) was used to measure intracellular ROS. Briefly, cells were pretreated with DCFH-DA probe (10 µM/L) for 20 min, 37 °C. After washing with 1× PBS three times, the fluorescence intensity was recorded on a Perkinelmer Enspire at Ex/Em = 488/525 nm, and the fluorescence imaging was performed by confocal microscopy (Zeiss LSM 800, Munich, Germany).

### 2.6. In Vitro H_2_O_2_ Concentration Measurement

The in vitro H_2_O_2_ measurement was performed by a hydrogen peroxide (H_2_O_2_) Content Assay Kit (#BC3590, SOLARBIO, Beijing, China) as per the manufacturer’s recommendations. Briefly, 20 μL aLDs were mixed with 20 μL ddH_2_O or 200μM H_2_O_2_ respectively. Subsequently, the mixture was incubated at 37 °C for 30 min, then the reagents were added sequentially according to the instructions. Absorbencies were measured at OD 415 on a microplate reader (Perkin Elmer, Waltham, MA, USA). The experiment was repeated three times.

### 2.7. Cell Counting Kit-8 (CCK8)

Hela cells activity after OA or PA treatment was detected by a Cell Counting Kit-8 (CCK8) kit (#A311-01, Vazyme, Nanjing, China). Briefly, cells were seeded at a density of 2 × 10^3^/well in 96-well plates. Then 10 μL CCK8 was added to each well and cells were cultured for another 2 h. The optical density at 450 nm was measured on a microplate reader (PerkinElmer, Rodgau, Germany).

### 2.8. Protein Expression and Purification

The Seipin and Plin5 genes were amplified from HepG2 cDNA and were cloned into the pET28a plasmid (MiaoLing Plasmid Sharing Platform). These plasmids were transformed into *E. coli* BL21 (DE3) and grown at 37 °C in LB to OD 600~0.6, and were then induced for 18 h with 0.6 mM isopropyl-D-thiogalactopyranoside (IPTG). An His-tag protein purification kit (#C600292-0001, Sangon, Shanghai, China) was used to purify the protein as per the manufacturer’s recommendations.

### 2.9. Antibodies

Rabbit polyclonal antibodies that were used included anti-VDAC1/Porin (#55259-1-AP, Proteintech, Wuhan, China), anti-CytoC (#A4912, Abclonal, Wuhan, China), and anti-GAPDH (#AC027, Abclonal, Wuhan, China). The following secondary antibodies were used: HRP (horseradish peroxidase)-labeled Goat Anti-Rabbit IgG (H + L; #AS014, Abclonal, Wuhan, China).

### 2.10. Isolation of Lipid Droplets (LDs)

LDs were purified from HepG2 cells after OA treatment by methods previously reported [19]. Briefly, cells were collected by adding 500 μL Buffer A (25 mm tricine, 25 mm sucrose, pH 7.8) containing 1 mm PMSF and incubated on ice for 30 min, and the cells were broken up using a homogenizer. The cells were then centrifuged at 16,000× *g* for 1 h at 4 °C, and the upper white LDs were collected and washed with 500 μL of buffer B twice.

### 2.11. Isolation of Mitochondria

A cell mitochondria isolation kit (#C3601, Beyotime Biotechnology, Nanjing, China) was utilized to isolate the mitochondrial fractions. Briefly, the cells were washed with cold PBS, and the cells were harvested by trypsin-EDTA solution. The cells were washed twice, collected by centrifugation, and then the supernatant was removed. Then, 1 mL mitochondrial isolation reagent (with 1 mM PMSF) was added, the cells were resuspended, and the suspension was incubated in an ice bath. Then, the cell suspension was transferred to a glass homogenizer of appropriate size, and homogenized approximately 10–30 times. The cell homogenate was centrifuged at 600× *g* and 4 °C for 10 min. Then, the supernatant was carefully transferred to another centrifuge tube and centrifuged for 10 min at 11,000× *g* and 4 °C. The precipitate was the isolated mitochondria. The supernatant collected was then centrifuged for 10 min at 12,000× *g* and 4 °C. The supernatant was the cytoplasmic protein without mitochondria.

### 2.12. Nanolipid Particle Lyophilization

We added the mixed final concentration of 5% (*w*/*v*) mannitol as the lyophilization protectant to the NLPs, dissolved and mixed the solution constituents, dispensed 2 mL of solution into a saline bottle, and the lyophilized sample was obtained after a prefreezing period of −40 °C for 4 h, a stabilization period of −15 °C for 30 min, a vacuum period of −15 °C for 4 h, a sublimation period of −10 °C for 18 h, 0 °C for 4.5 h and a final drying period of 20 °C for 3.5 h.

### 2.13. Fluorescence Microscopy

NLPs or purified LDs were incubated with BODIPY493/503 (1:1000 dilution), Flp (1:1000 dilution) or LipidTox red (1:1000 dilution) for 30 min at room temperature. Slides containing 4 μL NLPs or aLDs were sealed with an anti-fluorescent quenching solution (#P36961, ProLong™ Diamond Antifade Mountant, Invitrogen, Thermo Fisher, Waltham, MA, USA) for confocal microscopic observation (Zeiss LSM 800, Munich, Germany).

### 2.14. Cryo-Scanning Electron Microscopy (SEM)

First, the sample was frozen in liquid nitrogen, and then fractured under vacuum to reveal a fresh section. The water wrapped around the sample was sublimated and sprayed for conductivity. The sample was then placed on the cold stage of the SEM through the cryo-transfer system for observation.

### 2.15. Lipid Analysis by Thin-Layer Chromatography

Thin-layer chromatography followed previous experimental methods [22]. In short, the neutral lipids of the samples were separated first by hexane/diethyl ether/acetic acid (80:20:1, *v*/*v*/*v*). Then, the lipids were dried, and phospholipids were separated by chloroform/methanol/acetic acid/H_2_O (75:13:9:3, *v*/*v*/*v*/*v*). The samples were visualized by iodine vapor and analyzed by ImageJ.

### 2.16. Recruitment of Proteins to NLPs

Defined quantities of purified proteins were added to NLP preparations to a final volume of 100 μL. The mixture was gently vortexed and then incubated on ice or at room temperature for 1 h or longer. The NLPs were centrifuged at 20,000× *g* for 5 min, and the solution was removed for analysis. The remaining NLPs were then resuspended in 100 μL of buffer b and centrifuged again. The wash procedure was repeated three times to remove nonspecifically bound proteins. The washed NLPs were then observed by microscopy or for protein analysis.

### 2.17. Silver Staining

Silver staining was performed with the Fast Silver Stain Kit (#P0017S, Beyotime Biotechnology, Nanjing, China) following the manufacturer’s recommendations.

### 2.18. Statistical Analyses

All quantitative experiments were evaluated for statistical significance using the software GraphPad Prism v.5.0 (GraphPad Software, Inc. 7825 Fay Avenue, Suite 230 La Jolla, CA, USA), after verifying the normality of values and equivalence of variances. Data are displayed as the means  ±  S.D. Statistical tests were performed with two-tailed Student’s *t*-tests. A *p*-value < 0.05 was considered statistically significant.

## 3. Results

### 3.1. Formation of Nanolipid Particles and Their Characters

**Figure 1:**
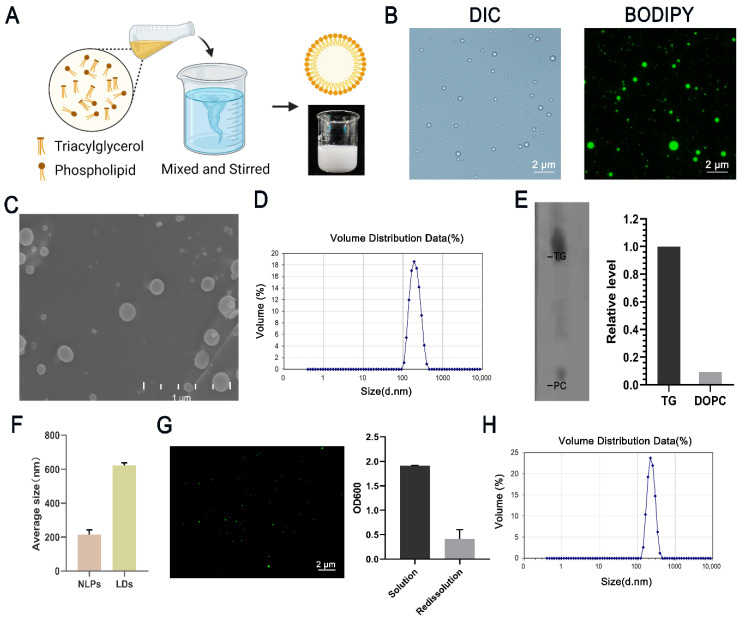
Preparation of NLPs and their properties. (**A**) Flow chart for the preparation of NLPs. Briefly, we used ethanol as a solvent to dissolve phospholipids and triglycerides by heating, added the organic phase to aqueous buffer, and stirred until the ethanol evaporated completely to form a solution of NLPs, which was a milky white liquid. The lower right side shows a picture after preparation. (**B**) DIC and fluorescence microscopy imaging of NLPs and BODIPY staining. Scale bars, 2 μm. (**C**) SEM imaging of NLPs. Scale bars, 1 μm. (**D**) Particle size distribution of NLPs. The average particle size was 215.3 nm. (**E**) TLC analysis of the DOPC/total lipids ratio of NLPs. The grayscale was analyzed by ImageJ. DOPC accounted for 8.5% of the total lipids, indicating the presence of a monolayer phospholipid structure on the NLPs. (**F**) HepG2 cells were treated with oleic acid, and LDs were extracted to measure their particle size compared to that of NLPs. The average size of HepG2 cell LDs was 623 ± 139.4 nm, and that of NLPs was 215.3 nm. *n* = 3, average ± SD. (**G**) The lyophilized powder of NLPs was redissolved in 4 mL of 0.2% Tween 80 solution, and the upper white lipid droplet band was collected after one centrifugation and then stained with BODIPY493/503 for observation. The optical density was measured. (**H**) Particle size distribution after rehydration of the freeze-dried powder. The average particle size was 233.5 ± 53.19 nm.

LDs are spherical and consist of a single phospholipid membrane and a neutral lipid core. To create this spherical lipid structure, we used phospholipids (DOPC) and neutral lipids (TAG) as raw materials. Briefly, we dissolved phospholipids and triglycerides by heating using ethanol as a solvent, added the organic phase to an aqueous buffer, and stirred until the ethanol evaporated completely to form a milky homogeneous solution, which was examined by light and phase contrast microscopy (Figure 1A). The results showed that almost all structures in the solution were spherical and stained positively for BODIPY493/503 (Figure 1B), indicating that these structures had neutral lipid nuclei. Circular structures could also be observed using scanning electron microscopy (Figure 1C). For better illustration, we refer to spherical lipid structures as nanolipid particles (NLPs). The average particle size of NLPs was 215.3 ± 72.45 nm (Figure 1D), and the zeta potential was −11.2 mV (Figure S1).

To further demonstrate the structure of the NLPs, we added rhodamine-labeled phosphatidylethanolamines to the organic phase and measured their fluorescence intensity using BODIPY493/503 staining, which showed that Rhod-PE was uniformly distributed in a circular shape and encapsulated neutral lipids (Figure S2). We extracted the lipids from the NLPs using the method in the literature, separated them using thin-layer chromatography, observed the lipids on thin-layer chromatography (TLC) plates by iodine vapor, and performed grayscale analysis using ImageJ, which showed a DOPC/total lipid ratio of 8.5%, indicating the presence of a monolayer phospholipid structure on the NLPs (Figure 1E). We then compared NLPs with LDs isolated from HepG2 cells. The average diameter of LDs from HepG2 cells was 623 ± 139.4 nm. The average size of NLPs was 215.3 nm (Figure 1F). After staining with various neutral lipid dyes [27], all NLPs showed spherical structures, which indicated that they both contained a neutral lipid core (Figure S3).

### 3.2. Stability and Utilization of Nanolipid Particles

We determined the stability of NLPs by incubating them at room temperature (RT) or 4 °C and examining them over time by optical density (OD) and particle size [22]. There were no significant changes in concentration or size and no significant changes in the morphology of the NLPs during the 7-day period, indicating that the NLPs were relatively stable (Figure S4).

To better preserve and utilize the LDs, we selected 5% mannitol as the lyophilization protectant. The results showed that the eutectic point was −5 °C (Figure S5). The lyophilized samples were a white loose porous powder with a smooth surface that was uniform and fine. After adding 2 mL of 0.2% Tween 80 solution, the powder could be redispersed quickly and was white and creamy after dispersion. Compared to newly prepared NLPs, the morphology of stored samples was normal after redissolution, and the number was reduced (Figure 1G). The average particle size was 233.5 ±53.19 nm (Figure 1H).

### 3.3. Lipid Composition Affects the Particle Size of NLPs

**Figure 2:**
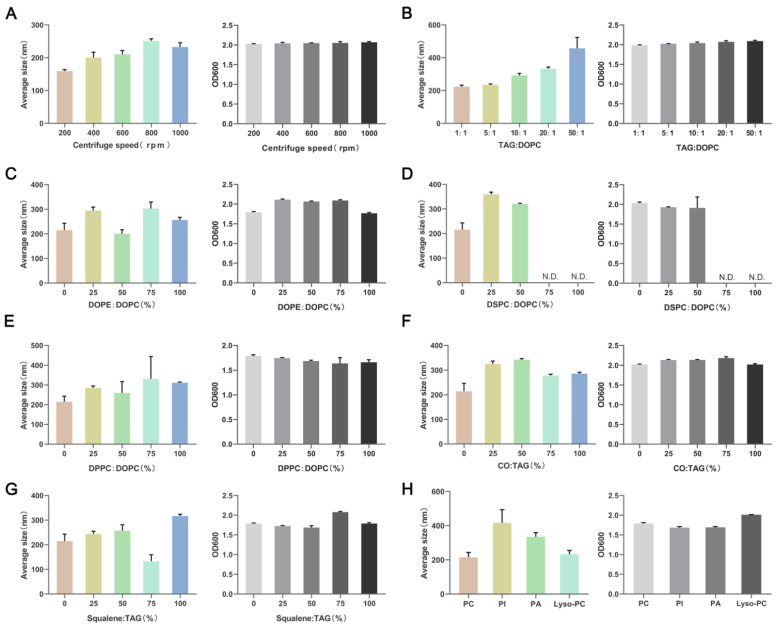
Concentration (OD 600) and size (average size) of lipid nanoparticles prepared under different conditions. (**A**) Concentration (OD 600) and size (average size) of NLPs under different stirring speeds. (**B**) Concentration (OD 600) and size (average size) of NLPs generated with different ratios of DOPC and TAG. DOPC, 1,2-di(9Z-octadecenoyl)-sn-glycero-3-phosphocholine; TAG, triacylglycerol. (**C**) Concentration (OD 600) and size (average size) of NLPs generated with different ratios of DOPE and DOPC. DOPE, 1,2-Dioleoyl-sn-glycero-3-phosphoethanolamine. (**D**) Concentration (OD 600) and size (average size) of NLPs generated with different ratios of DSPC and DOPC. DSPC, 1,2-Distearoyl-sn-glycero-3-phosphocholine. (**E**) Concentration (OD 600) and size (average size) of NLPs generated with different ratios of DPPC and DOPC. DPPC, 1,2-dipalmitoyl-sn-glycero-3-phosphocholine. (**F**) Concentration (OD 600) and size (average size) of NLPs generated with different ratios of CO and TAG. CO, Cholesterol. (**G**) Concentration (OD 600) and size (average size) of NLPs generated with different ratios of SQ and TAG. SQ, Squalene. (**H**) Concentration (OD 600) and size (average size) of NLPs generated with different ratios of multiple phospholipid fractions. The proportion of PI, PA and Lyso-PC to total phospholipids was 16.7%. PI, l-a-phosphatidylinositol; PA, phosphatidic acid; Lyso-PC, 1-hexanoyl-2-hydroxy-sn-glycero-3-phosphocholine.

LDs with small particle sizes are hard for observation in in vitro experiments. To obtain NLPs of different sizes, we systematically changed the two main factors in the preparation process, the magnetic stirrer speed and the ratio of phospholipids to TAG, and measured the yield and size of NLPs according to previous reports. The results showed that the particle size of NLPs increased when the stirring speed was increased, but when the speed was increased to 800 rpm, increasing the speed did not increase the particle size, and the yield was relatively stable without significant changes (Figure 2A). The ratio of TAG to DOPC also affected the yield and size of NLPs, and as the ratio increased, the size was increased, and the yield remained relatively stable (Figure 2B).

In living organisms, LDs have diverse phospholipid and lipid compositions, mainly including phosphatidylcholine, phosphatidylethanolamine, cholesterol, and squalene [28,29]. To better simulate the lipid composition of these droplets in organisms, we constructed NLPs with different lipid compositions. The phospholipid species were distinguished mainly by the head group, acyl chain saturation/unsaturation and length. First, we used DOPE to replace DOPC to simulate the effect of different head groups. Particle size was minimized at 50% DOPE content, and the yield decreased when the phospholipid composition was DOPE (Figure 2C). Both DOPC and DOPE are unsaturated phospholipids. Then we used saturated DSPC to replace unsaturated DOPC. The particle size of NLPs increased as the DSPC content increased with increasing DSPC content, and the yield decreased with increasing DSPC content. When the DSPC content was 75% and 100%, the solution showed obvious delamination after 5 min of standing, the upper white band was taken to observe that the NLPs were wrapped by many non-spherical structures, and pure NLPs could not be obtained, with a yield of 0. Therefore, DSPC could not be used as the main phospholipid component to prepare NLPs (Figure 2D). Next, we used DPPC with an acyl chain of palmitic acid (16C) instead of DOPC with an oleic acid chain (18C). With increasing DPPC content, the particle size of NLPs increased, and the yield decreased (Figure 2E).

Cholesterol is a common neutral lipid in LDs. By gradually replacing TAG with cholesteryl oleate (CO), the particle size of NLPs was maximized, and the yield increased when the cholesterol ratio was 50%. Further increasing the CO ratio did not increase the particle size of NLPs but even decreased it, and the yield was also slightly reduced (Figure 2F). Similarly, we added squalene (SQ) to the NLPs, and when the SQ ratio was increased, the particle size increased and the yield decreased; however, when the SQ concentration was increased to 75%, the particle size decreased and the yield increased (Figure 2G). Finally, we selected three other common components of LDs, phosphatidylinositol (PI), phosphatidic acid (PA) and lysolecithin (Lyso-PC), to test the production of NLPs, and the results reflected that all three of them could increase the particle size of NLPs, with PI having the strongest ability, while Lyso-PC could only slightly increase the particle size (Figure 2H). All these results indicate that the preparation process and lipid composition can greatly affect the yield and size of NLPs, and we can generate LDs of different sizes according to our needs.

### 3.4. Organelles and LD-Resident Protein Recruitment of NLPs

**Figure 3:**
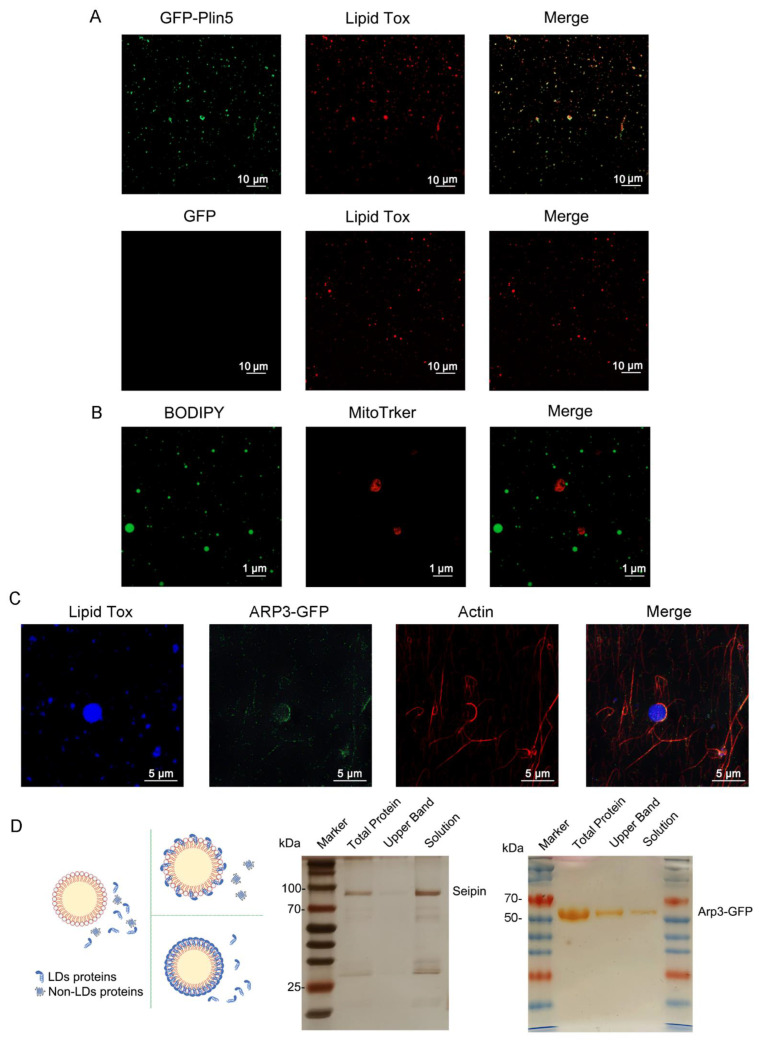
Organelles and specific protein recruitment of NLPs. (**A**) Plin5-GFP fusion proteins could be recruited to aLDs. Ten micrograms of GFP and Plin5-GFP fusion proteins were incubated with NLPs for 2 h. Then, the aLDs were stained with Lipid Tox and observed by confocal microscopy. Scale bars, 10 µm. (**B**) NLPs could be recruited to mitochondria. Extracted mitochondria were incubated with NLPs for 1 h. ALDs and mitochondria were stained with BODIPY493/503 and MitoTracker Red CMXRos, respectively, and subsequently observed by confocal microscopy. Scale bars, 1 µm. (**C**) NLPs could be recruited to microfilaments and surrounded by microfilaments in the shape of “hooks”. ARP3-GFP colocalized with microfilaments and was enriched at the contact site of all three proteins. NLPs, ARP3-GFP fusion protein and microfilaments were coincubated for 1 h. NLPs and microfilaments were stained with LipidTox and phalloidin, respectively, and subsequently observed by confocal microscopy. Scale bars, 5 µm. (**D**) Seipin was not recruited to NLPs. Ten micrograms of seipin were incubated with NLPs for 2 h. Arp3 was recruited to NLPs. Ten micrograms of Arp3 were incubated with NLPs for 2 h. The results were analyzed using silver staining. Only lipid droplet-resident/structure-like proteins were recruited to NLPs. Other types of proteins, such as multiple transmembrane proteins, were not recruited to NLPs.

LDs are highly dynamic organelles that are extensively involved in a variety of cellular metabolic pathways, such as glycolipid metabolism, oxidative stress, and autophagy. The performance of these functions depends on their surface proteins. In addition to the PAT family and lipid metabolism-related proteins, proteins with other functions, such as membrane transport or protein degradation, are also localized on LDs, so the recruitment of one or more of LDs proteins on NLPs is an important part of conferring their biological functions. In endogenous LDs, most proteins (especially perilipin family proteins) bind to LDs through amphipathic α-helices. Hydrophobic residues interact with a neutral core of LDs and anchor on the surface of LDs. Periplipin-5(Plin5) is an important protein in mammalian LDs that mediates the interaction between LDs and mitochondria. For better observation, we prepared a Plin5-GFP fusion protein with a GFP tag and coincubated it with NLPs at 37 °C for 1 h. We called these protein-binding NLPs artificial lipid droplets (aLDs), and the upper aLDs were collected by centrifugation and observed under a microscope. ALDs showed a punctate distribution of protein, and fusion protein aggregates were also visible (Figure 3A).

The interaction of LDs with organelles is also one of the important functions of LDs, among which the interaction with mitochondria is particularly important in glycolipid metabolism [30,31]. Based on this, HepG2 cell mitochondria were extracted and incubated with NPLs for 2 h. The NLPs were in contact with the siderophore mitochondria (Figure 3B), although it is unclear whether this contact was permanent or incidental. The binding of intracellular LDs to microfilaments enhances their interactions with other organelles [32]. Actin-related protein 3(ARP3) mediates the formation of branching actin networks in the cytoplasm, providing the apparatus for cell motility, which is usually located in microfilaments [33]. We found that after ARP3 overexpression, the protein localized to LDs (data not shown). Therefore, we coincubated the microfilaments, ARP3-GFP fusion protein and NLPs for 2 h. We stained the microfilaments with ghost pen loop peptide to observe the binding of the three. One end of the microfilament was a “hook” to the NLPs, and Apr3 was clearly colocalized with the microfilament and could be enriched at the contact site of the microfilament and NLPs (Figure 3C), similar to their intracellular localization. This “hook-like” structure may exist because intracellular microfilaments are longer and can interact with each other via motor proteins, while in vitro cultured microfilaments are shorter and can bind completely to aLDs.

We next investigated the specificity of recruitment of NLPs. Seipin protein plays a crucial role in LD generation, but it is localized in the endoplasmic reticulum and not in LDs [34,35]. We incubated 10 μg of Seipin protein with 50 μL of NLPs and then centrifuged the mixture. The mixture, the centrifuged upper lipid band and the centrifuged lower solution were taken separately for silver staining. As shown in the Figure 3D, Seipin was not detected in the upper band. This indicates that Seipin did not bind to the NLPs, which is consistent with the intracellular phenomenon and demonstrates the specificity of NLP protein recruitment. In contrast, ARP3-GFP (Figure 3D) could be observed in all three fractions, suggesting that the recruitment of the protein by the NLPs was specific. Lipoproteins in human blood also have a similar structure to LDs. Similarly, we coincubated the ApoB protein with NLPSs and detected the presence of this protein in both the upper and lower solution bands, indicating that the ApoB protein binds to NLPs and that this binding ability was saturable (Figure S6). The above protein binding experiments showed that the recruitment of proteins to NLPs was specific and that only LD resident/structure-like proteins could be recruited, while transmembrane proteins did not have such functions. Furthermore, the recruitment of NLPs to proteins was saturational, and proteins did not bind to NLPs without restriction. These data suggested that the prepared lipid nanoparticles could be used not only to study the function of LDs and their surface proteins but also as a tool and in vitro model for studying LD–organelle interactions.

### 3.5. Perilipin-Coated aLDs Promote Cells to Protect against the ROS

**Figure 4:**
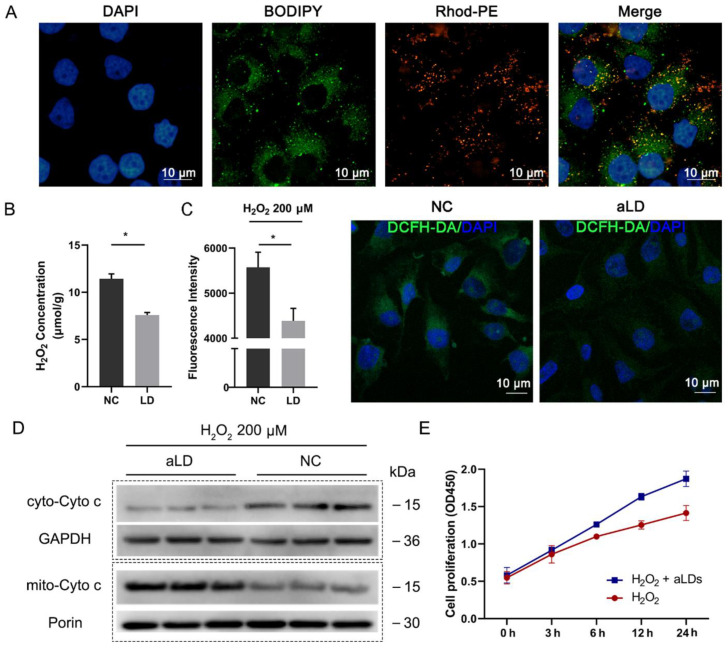
Cellular uptake and resistance to reactive oxygen species (ROS) of Plin5-Coated aLDs. (**A**) Confocal images representing the distribution of fluorescein-labeled LDs in Hela cells. Scale bar: 10 µm. (**B**) The aLDs were added to 200 μM H_2_O_2_ solution and the H_2_O_2_ content in the solution was measured after 30 min incubation. Data are presented as mean ± SD (*n* = 3). * *p* < 0.05. (**C**) Hela cells were incubated with aLDs for 4 h and then treated with 200 μM H_2_O_2_ for 12 h, and DCFH-DA was added to detect the Ros levels of the cells. NC: Hela cells were not incubated with aLDs. Data are presented as mean ± SD (*n* = 3). * *p* < 0.05. Scale bar: 10 µm. (**D**) Detection of cyto-C release from mitochondria after H_2_O_2_ treatment. (**E**) detection of cell proliferation of cell after H_2_O_2_ treatment.

Regulation of cellular stress is an important function of LDs. We first investigated the cellular uptake of aLDs. Consistent with a previous report [17], Rhod-PE labeled Plin5-coated aLDs co-localized with intracellular lipids (Figure 4A), which we used for subsequent experiments. Intracellular ROS are mainly present as H_2_O_2_, and we found that Plin5-coated aLDs could directly reduce the H_2_O_2_ concentration in vitro (Figure 4B). Subsequently, aLDs were co-incubated with Hela cells for 4 h and then 200 μM H_2_O_2_ was added to stimulate the cells. The results showed that the ROS level was significantly reduced after aLDs uptake (Figure 4C). Mitochondrial activity is an important factor affecting the level of oxidative stress in cells, and the level of cytochrome C (Cyto C) release from mitochondria to cytoplasm is the gold standard reflecting the level of mitochondrial activity and cellular oxidative stress. H_2_O_2_ treatment increased the release of Cyto C from mitochondria to the cytoplasm, while the cytoplasmic Cyto C level was reduced by the addition of aLDs (Figure 4D). Implying that cells enhanced stress resistance after uptake of aLDs, this elevation was also reflected in cell proliferation capacity (Figure 4E).

### 3.6. Perilipin-Coated aLDs Promote Protection by Cells against Lipotoxicity

**Figure 5:**
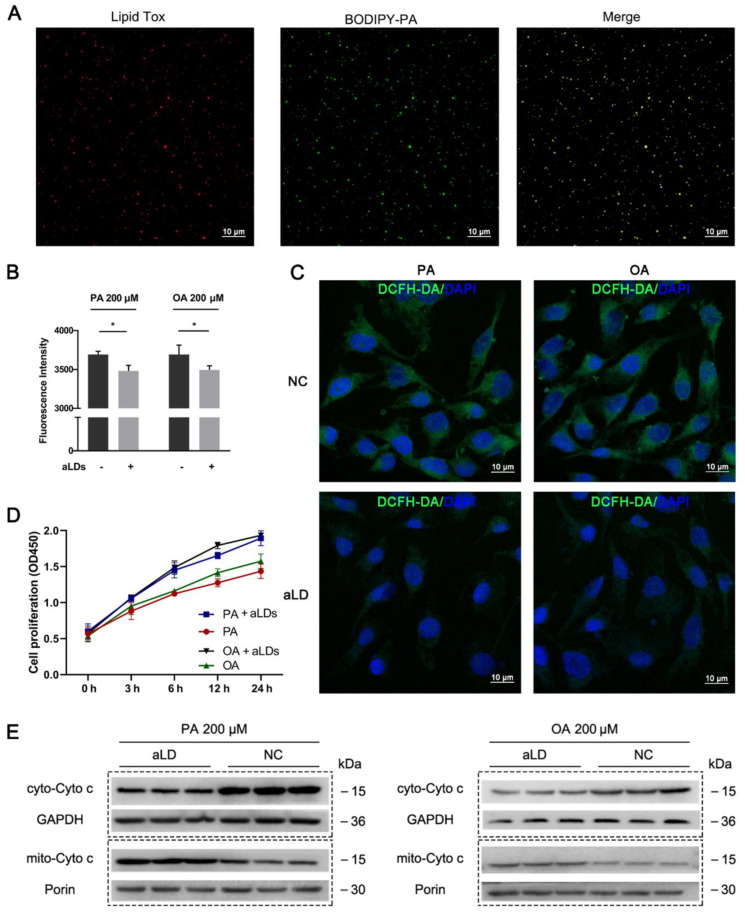
Fatty acid absorption and alleviation of lipotoxicity. (**A**) Detection of BODIPY-PA Localization. Scale bar: 10 µm. (**B**) Detection of ROS after PA/OA treatment in Hela cell. Data are presented as mean ± SD (*n* = 3). * *p* < 0.05. Scale bar: 10 µm. (**C**) Observation of ROS after PA/OA treatment in Hela cell. NC: Hela cells were not incubated with aLDs. Scale bar: 10 µm. (**D**) Detection of cell proliferation of cell after PA/OA treatment. (**E**) Detection of cyto-C release from mitochondria after PA/OA treatment.

Excess fatty acids can trigger cellular lipotoxicity and cause cellular damage. After we co-incubated BODIPY-labeled palmitic acid (PA) with aLDs, a clear co-localization could be observed (Figure 5A), indicating that aLDs have the ability to absorb fatty acids. Next, we blocked cellular lipid synthesis by diacylglycerol O-acyltransferase 1 (DGAT1, A922500) and diacylglycerol O-acyltransferase 2 (DGAT2, PF-06424439) inhibitor firstly. After incubating the cells with aLDs for 4 h, the cells were treated with 200 μM PA and OA, respectively. The results showed that the access of aLDs to cells reduced ROS caused by both OA and PA (Figure 5B,C), decreased Cyto C transfer from mitochondria to cytoplasm (Figure 5D) and improved cell proliferation (Figure 5E). This indicated that aLDs had consistent biological activity with endogenous LDs and reduced lipotoxicity caused by excess lipids. This demonstrates that LDs are themselves factors that regulate cellular stress and have the ability to regulate lipid content.

## 4. Discussion

Beyond energy storage, LDs play an important role in cellular oxidative stress resistance. Many studies showed that the elevated ROS content was accompanied by the increased cellular LDs number. The evidence indicated that LDs helped cells to protect against oxidative stress by reducing oxidative damage to cell membranes and maintaining the homeostasis of the endoplasmic reticulum [36]. The turnover of LDs during stress was regulated by adenosine 5’-monophosphate (AMP)-activated protein kinase (AMPK) [37] and mTORC1 [38] pathways, which control lipolysis and synthesis of FA and glycerol esters. Moreover, a recent study identified an interesting function of LDs in cleaning the harmful proteins such as apoptosis regulator (BAX) and BCL2 Like 1(BCL-XL) from the out-membrane of mitochondria which was impaired by excess ROS [39]. Therefore, LDs are essential in reducing the injury of phospholipids membrane and mitochondria.

Excess fatty acid can lead to lipotoxicity and result in cellular damage. LDs are the center regulator of them. LDs act as reservoirs by reducing lipid fluctuations either by esterify excess fatty acids. However, the mechanism of the alleviating effect of LDs on lipotoxicity is not well understood. The analysis of this mechanism allows us to understand more clearly the function of LDs. However, the highly dynamic LDs pose a great challenge to this research. Therefore, it is important to develop a simple and convenient in vitro model system. Previously, the Liu team prepared artificial fat droplets using vortices, which established the method of preparation and utilization of LDs, but this method produced a large number of non-spherical structures in the mixture, and purer artificial fat droplets could only be obtained by two centrifugations [22].

In our study, high-purity aLDs can be obtained by a simple preparation procedure. We also provide a preservation method by preparing NLPs as a lyophilized powder that can be used after redissolution. The prepared aLDs are protein-free, and are able to take advantage of specific LD functions. This pure and less disturbing property is not available for in vivo studies. Our study found that Plin5-coated aLDs reduced H_2_O_2_ levels and absorbed free fatty acids in vitro. This suggests that LDs may play a direct anti-stress role. Subsequently, aLDs were absorbed and helped cells to effectively resist stress and mitigate lipotoxicity. This not only indicates that aLDs have the same biological function as endogenous LDs, but also shows that LDs themselves are factors that regulate cellular oxidative stress independent of their surface proteins.

To better simulate the lipid components of NLPs, we selected a variety of lipid components for experiments. Clearly, different lipid components have a great influence on the formation of NLPs, showing similarity with the reported results [22]. Through horizontal comparison of various lipid components, it was found that DSPC, DOPE, squalene and CO increased the particle size of NLPs, which is similar to the results of Ben [40]. He simulated the state of LD budding in vitro and found that these lipids can form larger LDs during budding. Similarly, the length of the phosphatidyl chain is also an important factor. The main difference between DSPC and DPPC lies in the length of the acyl chain. The palmitoyl chain of DPPC is shorter than the stearyl chain. The particle size of short-chain phospholipids is smaller than that of DSPC. This might be associated with the phospholipid packing on the LDs surface. The phospholipids with long acyl chains resulted in a higher degree of packing, leading to lower surface tension. LDs became larger when the surface tension became low, and otherwise LDs became smaller [41]. The effects of these lipids may be similar to the physiological laws followed in cells. The aLDs we prepared were similar to endogenous LDs in terms of lipid components and size, which laid the foundation for in vitro studies of LDs.

ALDs can help cells to resist stress. This caused us to consider that aLDs might become a treatment for acute stress in the future. In our experiments, the apolipoprotein (ApoB), the main component of lipoproteins in the blood, can also be recruited to nanoparticles. This provides the possibility for the use of NLPs as drug carriers. Compared with traditional drug carriers, the main components of NLPs are phospholipids and triglycerides, which are more natural, more stable, and do not easily cause intense immune rejection. It has been shown that LDs can promote drug accumulation [16]. Labeled bedaquiline has been shown to accumulate primarily in host cell LDs and enhance antibacterial efficacy [15]. Isolated LDs can deliver anti-cancer drugs and significantly inhibit tumor growth. In our study, NLPs mimicked the basic structure of LDs and provide a controlled size distribution regulation method and demonstrated their stability and biocompatibility. More importantly, NLPs can be stably preserved after being made into lyophilized powder using our method. This drug-delivery system based on artificial organelles can improve existing cell-based therapies to the subcellular level, provide new insights into drug delivery, and holds promise for clinical applications. Oxidative stress is generated by inflammatory processes such as cardiovascular diseases, autoimmune diseases, and cancer. The antioxidant capacity can be improved by exogenous injection of aLDs [42].

## 5. Conclusions

In summary, we have developed a new in vitro research model of aLDs. The prepared aLDs are of high purity and can be preserved for a long time by freeze-drying. The NLD lipid composition and recruitment of different proteins or organelles can be controlled for functional research. Cellular uptake of aLDs can resist ROS and mitigate lipotoxicity, with potential biological and medical value.

## Data Availability

Not applicable.

## Supplementary Materials

The following are available online at https://www.mdpi.com/article/10.3390/nano12040672/s1, Table S1: List of abbreviations. Figure S1: Zeta potential of nanolipid particles. Figure S2: The fluorescence distribution of Rhod-PE. Figure S3: Staining of NLPs using different dyes. Figure S4: Stability of nanolipid particles. Figure S5: Eutectic point curve of nanolipid particles. Figure S6: ApoB was recruited to nanolipid particles.
