# Supplementary material for: Artificial Lipid Droplets: Novel Effective Biomaterials to Protect Cells against Oxidative Stress and Lipotoxicity
Source: Nanomaterials (Basel). 2022 Feb 17;12(4):672. doi: 10.3390/nano12040672 (PMC8879118; doi:10.3390/nano12040672)
Supplement: Supplementary file 1 [file nanomaterials-12-00672-s001.zip › nanomaterials-1507437-supplementary.pdf]

nanomaterials

Supplementary materials
Artificial Lipid Droplets: Novel Effective Biomaterials to Protect Cells against Oxidative Stress and Lipotoxicity

Pengxiang Zhao [1], Yi Jin [1], Xiang Wu [1], Jin Huang [1], Lupeng Chen [1], Yanjie Tan [1], Hong Yuan [2], Jian Wu [1] and Zhuqing Ren [1,3,*]

[1] Key Laboratory of Agriculture Animal Genetics, Breeding and Reproduction of the Ministry of Education, College of Animal Science, Huazhong Agricultural University, Wuhan 430070, China; pengxiang@webmail.hzau.edu.cn (P.Z.); Jinhongyi@mail.hzau.edu.cn (Y.J.); Wx1078724218@163.com(X.W.); huangjin@webmail.hzau.edu.cn (J.H.); chenlupeng@webmail.hzau.edu.cn (L.C.); 620036@sdnu.edu.cn (Y.T.); wujian@mail.hzau.edu.cn (J.W.)
[2] Key Laboratory of Pesticide and Chemical Biology of Ministry of Education, College of Chemistry, Central China Normal University, Wuhan 430079 China; yuanhong@mail.ccnu.edu.cn
[3] Hubei Hongshan Laboratory, Hubei province, China
* Correspondence: renzq@mail.hzau.edu.cn

Table S1. List of abbreviations.

abbreviation	full name
LD	Lipid Droplets
ER	Endoplasmic Reticulum
ROS	Reactive Oxidative Species
NLPs	Nanolipid Particles
aLDs	Artificial Lipid Droplets
PA	Palmitic Acid
DOPC	1,2-Di(9z-Octadecenoyl)-Sn-Glycero-3-Phosphocholine
DOPE	1,2-Di(9z-Octadecenoyl)-Sn-Glycero-3-Phosphoethanolamine
DSPC	1,2-Dioctadecanoyl-Sn-Glycero-3-Phosphocholine
DPPC	1,2-Dipalmitoyl-Sn-Glycero-3-Phosphocholine
TAG	Triacylglycerol
CO	Cholesteryl Oleate
PI	Phosphatidylinositol
ATGL	Adipose Triglyceride Lipase
MUFA	Monounsaturated Fatty Acids
SIRT1	Sirtuin 1
PGC-1α	Pparg Coactivator 1 Alpha
LasA	Lasonolidea
LDAH	Lipid Droplet Associated Hydrolase
MLDS	Microorganism Lipid Droplet Small Protein
COPI	Coat Complex Subunit Beta 1
GUV	Giant Unilamellar Vesicle
APOB	Apolipoprotein B
DGAT1	Diacylglycerol O-Acyltransferase 1
DGAT2	Diacylglycerol O-Acyltransferase 2
TLC	Thin-Layer Chromatography
OD	Optical Density
SQ	Squalene

Lyso-PC	Lysolecithin
ARP3	Actin-Related Protein 3
BAX	Bcl2 Associated X
BCL-XL	Bcl2 Like 1
NC	Negative Control

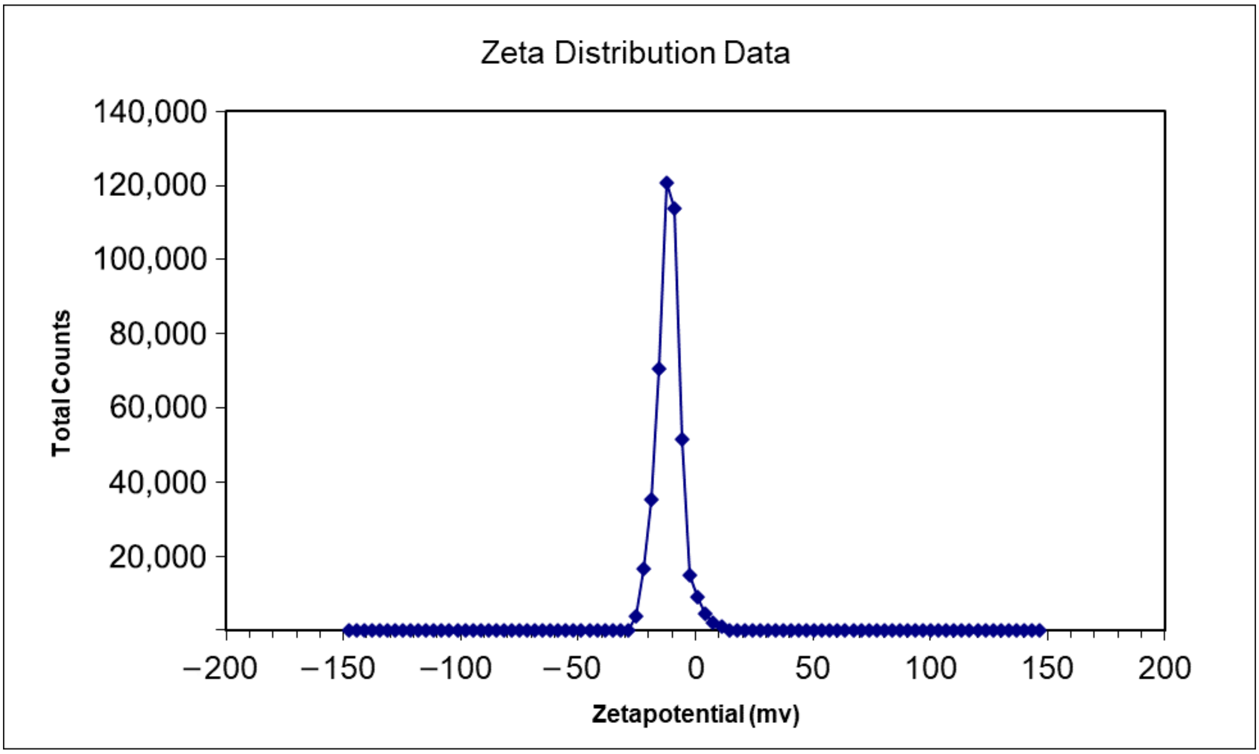

Figure S1. Zeta potential of nanolipid particles.

The zeta potential was −11.2 mV.

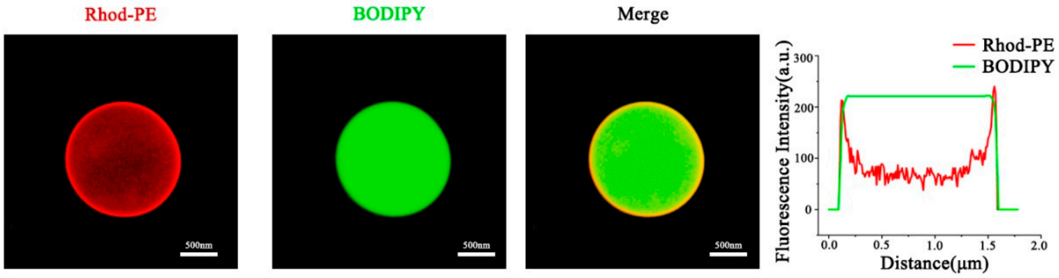

Figure S2. The fluorescence distribution of Rhod-PE.

The three images on the left show representative fluorescence images of the same areas of observation of NLPs (Rhod-labeled DOPE lipids in red), which were stained using green fluorescence, and the two images superimposed together show that the phospholipids were uniformly distributed in a ring-like pattern around the periphery of the neutral lipids. On the far right is the fluorescence intensity curve, where the phospholipids were clearly positioned at the periphery of the neutral lipids. Scale bars, 500 nm.

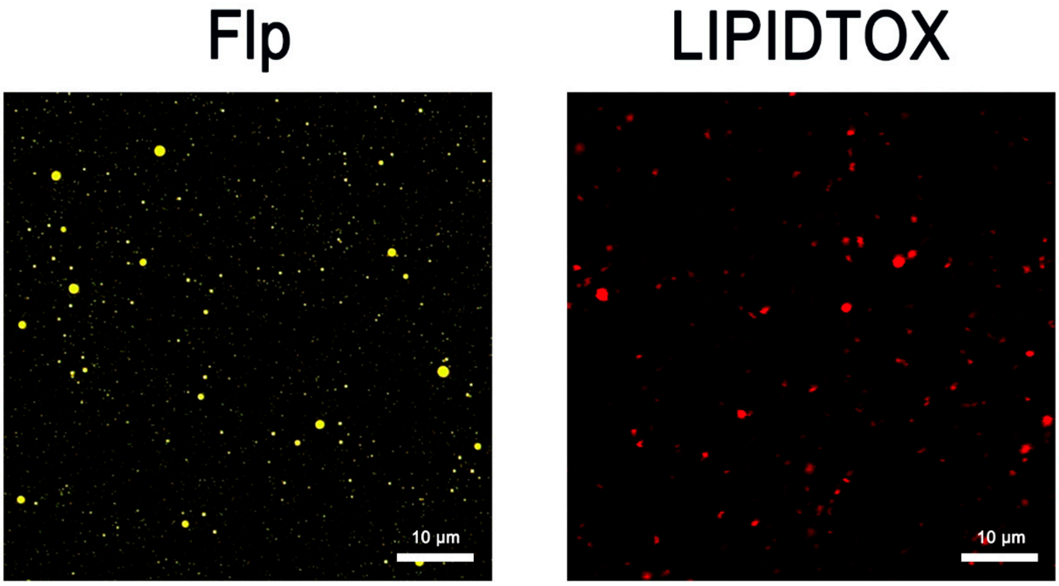

Figure S3. Staining of NLPs using different dyes.

Staining of NLPs using different dyes. Staining of NLPs using two neutral lipid-specific dyes, Flp and Lipid Tox, and subsequent confocal microscopy observations. Scale bars, 10 μm.

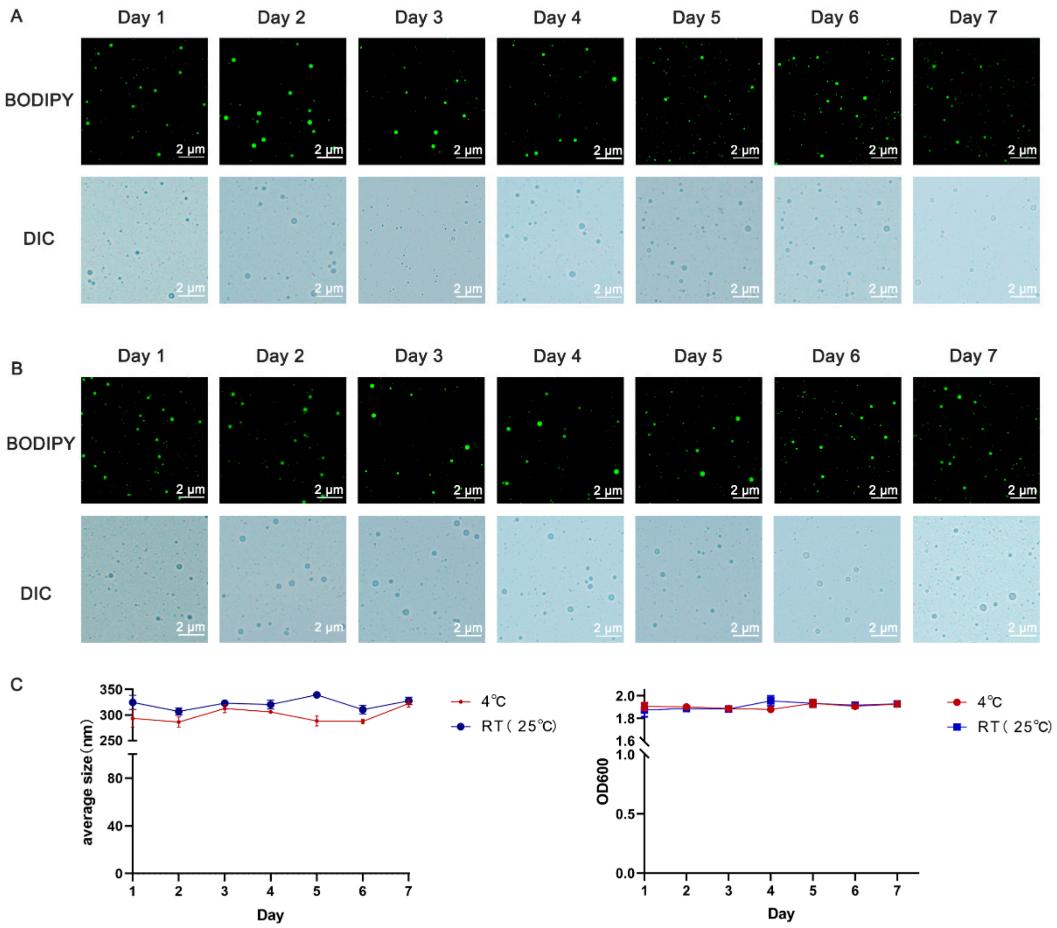

Figure S4. Stability of nanolipid particles. (**A**). Nanolipid particles were stored at 4 °C for 7 days, and their morphology was observed daily with DIC. Results of staining with BODIPY493/503 were observed by confocal microscopy. Scale bars, 2 μm. (**B**). Nanolipid particles were stored at RT (25 °C) for 7 days, and morphology was observed daily with DIC, and BODIPY493/503 staining was observed by a confocal microscope. Scale bars, 2 μm. (**C**). Size (average size) and Concentration (OD 600) of NLPs under 4℃ and RT (25℃) during 7days.

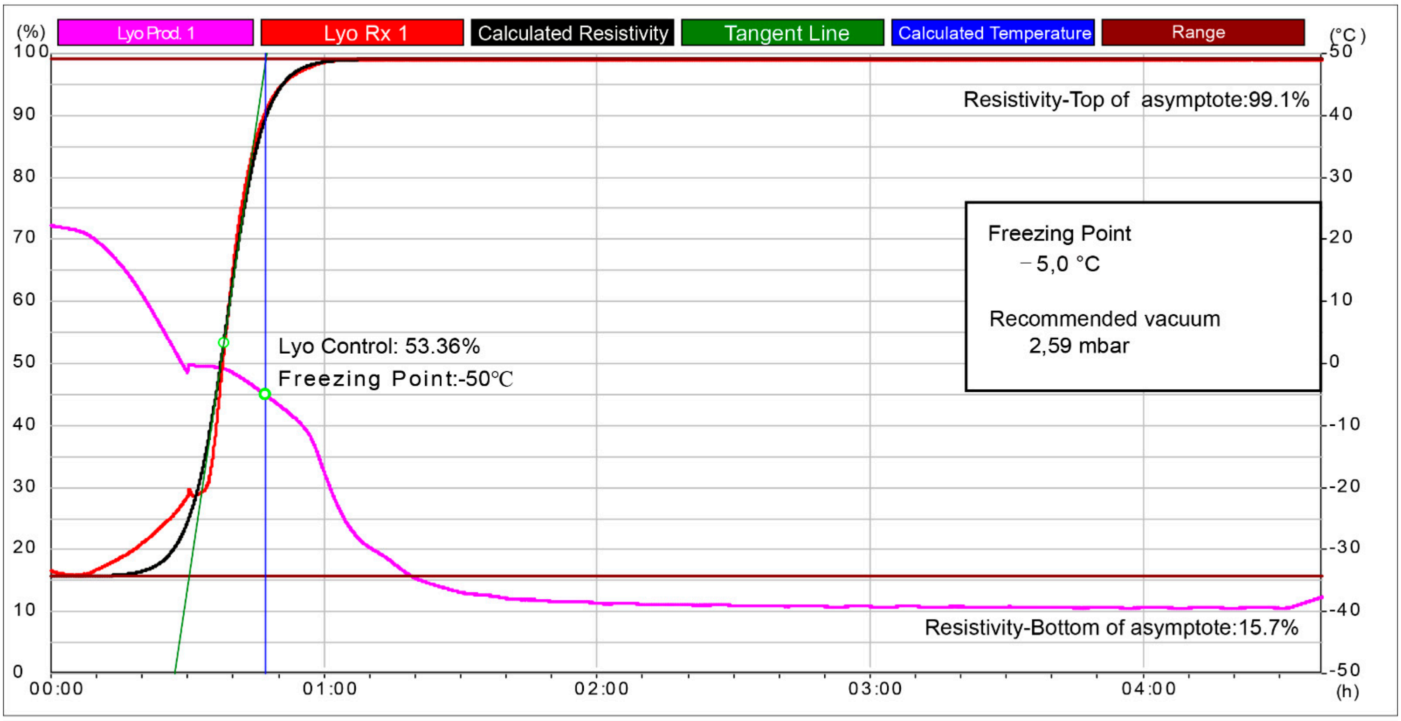

Figure S5. Eutectic point curve of nanolipid particles.

The freezing point of NLPs was −5.0℃.

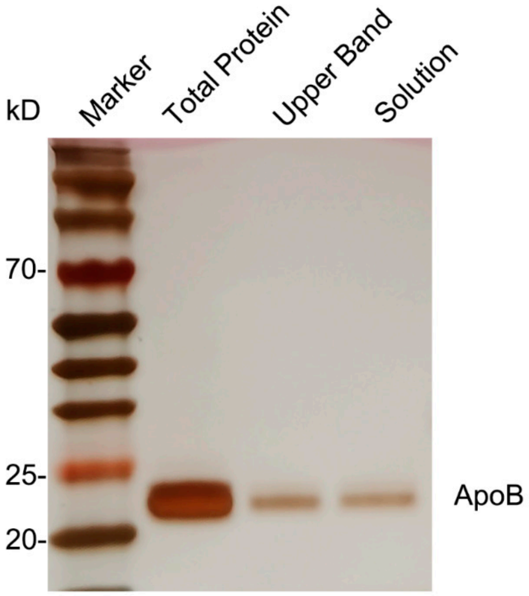

Figure S6. ApoB was recruited to nanolipid particles.

Ten micrograms of ApoB was incubated with nanolipid particles for 2 h. The results were analyzed using silver staining.
